# Cognitive functions and their correlates in people with psychosis: are there gender differences?

**DOI:** 10.1192/j.eurpsy.2023.116

**Published:** 2023-07-19

**Authors:** L. Fusar-Poli

**Affiliations:** Department of Brain and Behavioral Sciences, University of Pavia, Pavia, Italy

## Abstract

**Abstract:**

Impairments in social and non-social cognition are common in psychosis and may be sparsely present even before the onset of the disorder. Genetic and environmental influences have been linked to cognitive dysfunctions, which, in turn, may significantly impact the real-world functioning of people with psychosis. The role of gender in determining the interplay between cognitive skills, risk factors, and outcomes has been relatively unexplored. Nevertheless, identifying putative gender differences in cognitive functions and their correlates may favor the identification of individualized prevention and treatment strategies.

**Disclosure of Interest:**

None Declared

